# Application of the #Enzian classification for endometriosis on MRI: prospective evaluation of inter- and intraobserver agreement

**DOI:** 10.3389/fmed.2023.1303593

**Published:** 2023-11-17

**Authors:** Sebastian Harth, Hasan Emin Kaya, Felix Zeppernick, Ivo Meinhold-Heerlein, Jörg Keckstein, Selcuk Murat Yildiz, Emina Nurkan, Gabriele Anja Krombach, Fritz Christian Roller

**Affiliations:** ^1^Department of Diagnostic and Interventional Radiology, Justus Liebig University Giessen, Giessen, Germany; ^2^Department of Radiology, School of Medicine, Görükle Campus, Bursa Uludağ University, Bursa, Türkiye; ^3^Department of Gynecology and Obstetrics, Justus Liebig University Giessen, Giessen, Germany; ^4^Endometriosis Clinic Dres. Keckstein, Villach, Austria; ^5^Department of Obstetrics and Gynecology, Medical University Ulm, Ulm, Germany; ^6^SEF, Westerstede, Germany

**Keywords:** pelvis, deep infiltrating endometriosis, endometrioma, magnetic resonance imaging, interobserver variability

## Abstract

**Objectives:**

The purpose of this investigation was to evaluate the inter- and intraobserver variability of the updated #Enzian classification of endometriosis on MRI and to evaluate the influence of reader experience on interobserver concordance.

**Methods:**

This was a prospective single-center study. All patients were included who received an MRI of the pelvis for evaluation of endometriosis between March and July 2023 and who have provided written informed consent. Images were reviewed independently for endometriosis by three radiologists, utilizing the MRI-applicable categories of the #Enzian classification. Two radiologists had experience in pelvic MRI and endometriosis imaging. One radiologist had no specific experience in pelvic MRI and received a one-hour briefing beforehand.

**Results:**

Fifty consecutive patients (mean age, 34.9 years ±8.6 [standard deviation]) were prospectively evaluated. Interobserver agreement was excellent for diagnosis of deep infiltrating endometriosis (Fleiss’ kappa: 0.89; 95% CI 0.73–1.00; *p* < 0.001) and endometriomas (Fleiss’ kappa: 0.93; 95% CI 0.77–1.00; *p* < 0.001). For the experienced readers, interobserver agreement in the assessment of compartments A, B and C was excellent (κ_w_ ranging from 0.84; 95% CI 0.71–0.97; *p* < 0.001 to 0.89; 95% CI 0.82–0.97; *p* < 0.001). For the pairings of the experienced readers to the reader without specific experience in pelvic MRI, agreement was substantial to excellent (κ_w_ ranging from 0.64; 95% CI 0.44–0.85; *p* < 0.001 to 0.91; 95% CI 0.84–0.98; *p* < 0.001). Intraobserver variability was excellent for compartments A, B and C (κ_w_ ranging from 0.85; 95% CI 0.73–0.96; *p* < 0.001 to 0.95; 95% CI 0.89–1.00; *p* < 0.001).

**Conclusion:**

With sufficient experience, the #Enzian classification enables the achievement of excellent inter- and intraobserver agreement in MRI-based diagnosis of deep infiltrating endometriosis and endometriomas.

## 1 Introduction

MRI is widely used and recommended in the diagnosis of deep infiltrating endometriosis (DIE) (1–3). Multiple attempts have been made to describe the extent of endometriosis, but to date no universally accepted classification system exists (4–6). In 2021, the #Enzian classification has been published to provide a comprehensive resource for the description and staging of endometriosis (7). The classification has been created to overcome limitations of the Enzian classification (established in 2003 and revised in 2011) (8) and the revised American Society for Reproductive Medicine classification of endometriosis (rASRM) and allows a complete description of superficial and deep infiltrating endometriosis, ovarian endometriosis, and uterine adenomyosis (9). Application of the #Enzian classification is intended for both surgical and diagnostic specialties and aims to enable communication and documentation of findings of surgery, ultrasound, and MRI clearly and objectively.

Reports on the applicability of the upgraded #Enzian classification for MRI examinations are promising (10, 11), but data on inter- and intraobserver variability are scarce. In one retrospective study, Manganaro et al. have reported overall good interobserver agreement (Cohen’s kappa 0.73) of the #Enzian classification when applied to MRI (12). However, further studies are warranted as existing data are limited. A prospective evaluation of the interobserver variability of the updated #Enzian classification on MRI has not been reported yet. Furthermore, a detailed analysis of all MRI-applicable categories of the classification is pending, including evaluations of the assignment of lesions to the left and right body side (categories B, O) and evaluations of ordinal scaled data. Additionally, the evaluation of the influence of reader experience on interobserver agreement is of interest. Saba et al. found a significant increase in the accuracy of endometriosis diagnosis on MRI with experience (13), but studies on the #Enzian classification in this regard are not yet available.

The purpose of this investigation was therefore to evaluate the inter- and intraobserver variability of the MRI-applicable categories of the updated #Enzian classification and to evaluate the influence of reader experience on interobserver concordance.

## 2 Materials and methods

Ethical approval for this prospective, non-interventional study was obtained from the local institutional review board (IRB) and written informed consent from all participants was received (German Clinical Trials Register ID DRKS00031403).

### 2.1 Patients

We prospectively included 50 consecutive patients aged 18 years or older who were scheduled to undergo a pelvic MRI scan for suspected endometriosis at our tertiary care center from March 2023 to July 2023. The indications for the MRI examinations were established after clinical gynecological examination and transvaginal ultrasound. Exclusion criteria were pregnancy and inability or unwillingness to consent. MRI scans were conducted at two 1.5 Tesla scanners (Avanto, Siemens Healthcare, *n* = 40; Espree, Siemens Healthcare, *n* = 10). No adverse events were encountered in the course of the MRI examinations. All patients have provided written informed consent.

### 2.2 MRI protocol for endometriosis

Patients were examined with an MRI protocol that is used in clinical practice and includes commonly recommended sequences for the evaluation of endometriosis (14, 15): Axial, sagittal, and coronal T2-weighted FSE (fast spin echo), axial T1-weighted FSE and axial fat-suppressed T1-weighted FSE.

According to current guidelines, MRI examinations were scheduled independently of the menstrual cycle (14). The preparation of the patients included rectal contrast with water and vaginal contrast with ultrasound gel when consent was given (48/50 and 44/50, respectively) (14, 16). An anti-peristaltic agent was administered in most patients (intravenous hyoscine butylbromide 20 mg, Carinopharm GmbH, 48/50). To achieve moderate filling and good assessability of the urinary bladder, care was taken to ensure that patients did void their bladder approximately 1 h before the examination and did not void their bladder afterwards until the completion of the MRI examination.

Intravenous administration of gadolinium based contrast agents (GBCAs) was performed optionally, depending on additional questions and the findings of the non-contrast images (11). For 40/50 (80.0%) patients, it was decided that contrast administration was not necessary. In 10/50 (20.0%) patients, GBCAs were administered (Gadoteridol, ProHance, 0.1 mmol/kg, Bracco Imaging s.p.a.) for the following reasons: indeterminate ovarian lesion (5/50), suspicion of pelvic venous congestion syndrome (3/50), indeterminate uterine mass (2/50).

### 2.3 MRI image analysis

All images were reviewed independently by three senior radiologists from two different medical centers on Picture Archiving and Communication System (PACS) workstations. Two radiologists (S.H., F.C.R.) had experience in pelvic MRI and endometriosis imaging (7 and 5 years, respectively). The third radiologist (H.E.K.) was a musculoskeletal radiologist without specific experience in pelvic MRI. The latter reader received a one-hour briefing by the radiologist with 7 years’ experience with the following content: demonstration of the #Enzian classification based on the publications by Keckstein et al. (7) and Harth et al. (3); discussion of four exemplary cases that were not drawn from the collective of the present study (Case 1: #Enzian(m) A2, B2/2, C3, FA(external), FU(l); Case 2: #Enzian(m) A1, B2/2, C1, O1/0; Case 3: #Enzian(m) A1, B2/3, FA(external), FI(Sigma); Case 4: No endometriosis); discussion of different forms (internal, external) and diagnostic criteria of adenomyosis uteri (17); discussion of uterine contractions as mimickers of adenomyosis (18). Figures from the 2021 publication by Keckstein et al. and the 2023 publication by Harth et al. were made available to guide all readers (3, 7). Images of cases used for training were not included in later image analysis.

**Figure 1:**
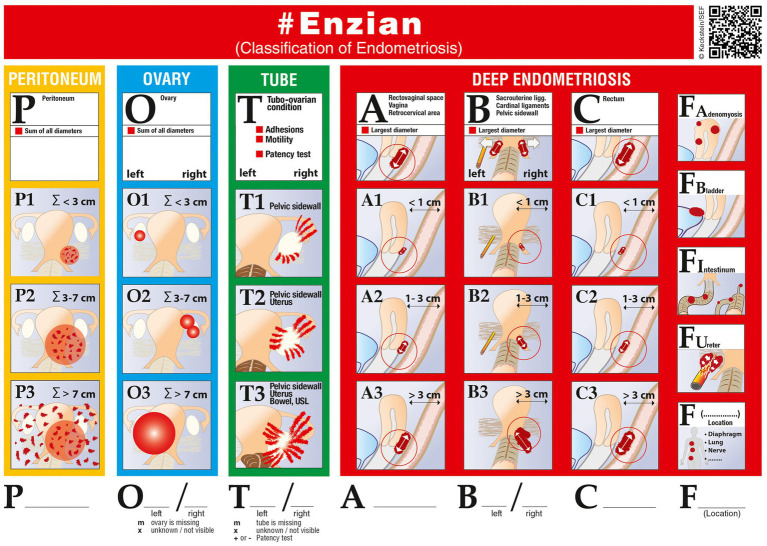
The #Enzian classification of endometriosis (reproduced with permission of J. Keckstein/Scientific Endometriosis Foundation, https://www.endometriose-sef.de/aktivitaeten/klassifikation-enzian/).

The radiologists evaluated each MRI for evidence of endometriosis independently. For this purpose, the categories of the #Enzian classification applicable in MRI were taken into account (Figure 1) (10): compartment A, comprising the rectovaginal space, the vagina, and the retrocervical area; compartment B with individual assessments of the right and the left side, comprising the sacrouterine ligaments, the cardinal ligaments, and the pelvic sidewall; compartment C (rectum); organ O (ovary) with individual assessment of the right and the left side; category FA (adenomyosis); organ FB (bladder); organ FI (intestinum); organ FU (ureter); and category F(…), covering other anatomic sites. For compartments A, B, and C, the size of lesions was measured and graded according to the increments proposed in the #Enzian classification (1: <1 cm, 2: 1–3 cm, 3: >3 cm). The diameters of endometriomas were added for each body side and graded accordingly (1: ∑ < 3 cm, 2: ∑ 3–7 cm, 3: ∑ > 7 cm). #Enzian categories P and T were omitted from the evaluation, as applicability on MRI is limited (10).

Three to seven months after completion of the first assessment, all 50 cases were assessed again by one of the experienced readers (S.H.) in a blinded evaluation without access to MRI reports, clinical data, or the results of the initial evaluation.

### 2.4 Statistical analysis

Statistical analyses were performed utilizing IBM SPSS Statistics 29.0.

Sample size estimation was performed to detect statistically significant Cohen’s kappa coefficients (κ) (*p* ≤ 0.05) on dichotomous and dichotomized variables, following the recommendations by Sim and Wright (19): With 80% power, expecting a proportion of positive ratings in the range of 35–45% (3, 11), expecting a minimum value for Cohen’s kappa coefficient of 0.60, and assuming the null-hypothesis kappa to be 0.00, a minimum sample size of 22 was determined for a two-tailed-test.

Cohen’s kappa coefficients (κ) were computed for dichotomous variables (DIE all locations, FA, FB, FI, F(…), FU) and dichotomized variables (O both sides, O left side, O right side, A, B both sides, B left side, B right side, C) to assess agreement for pairs of two raters (reader 1 and 2, reader 1 and 3, reader 2 and 3, reader 1 and 1). For ordinal scaled variables (O0-3 left side, O0-3 right side, A0-3, B0-3 left side, B0-3 right side, C0-3), quadratic weighted kappa coefficients (κ_w_) were computed.

To assess agreement of all three raters, Fleiss’ kappa was calculated for dichotomous variables (DIE all locations, FA, FB, FI, F(…), FU) and dichotomized variables (O both sides, O left side, O right side, A, B both sides, B left side, B right side, C), and Kendall’s W was calculated for ordinal scaled variables (O0-3 left side, O0-3 right side, A0-3, B0-3 left side, B0-3 right side, C0-3).

Reader agreement was assessed using the following range definitions of kappa values: 0.81 and 1.00, excellent (‘almost perfect’); 0.61–0.80, substantial; 0.41–0.60, moderate; 0.21–0.40, fair; 0.00–0.20 slight (20).

## 3 Results

Fifty consecutive patients (mean age, 34.9 years ±8.6 [standard deviation]) were prospectively evaluated for endometriosis by three readers on MRI, utilizing the #Enzian classification. Table 1 summarizes the characteristics of the study population.

**Table 1:** Patient demographics and clinical characteristics.

Characteristic	*N*/total (%) unless shown otherwise
Age (years), mean ± SD, range	34.9 ± 8.6, 18–55
BMI (kg/m^2^) ± SD	24.7 ± 4.8
Prior abdominal surgery	31/50 (62.0)
Laparoscopy for endometriosis	21/50 (42.0)
Cesarean section	9/50 (18.0)
Appendectomy	6/50 (12.0)
Laparoscopy for ovarian mass	4/50 (8.0)
Laparoscopy for adhesions	4/50 (8.0)
Total laparoscopic hysterectomy	4/50 (8.0)
Inguinal hernia repair	2/50 (4.0)
Rectum resection with anastomosis	2/50 (4.0)
Psoas hitch	2/50 (4.0)
Laparoscopic myomectomy	2/50 (4.0)
Neurostimulator implantation	2/50 (4.0)
Other surgical procedures	7/50 (14.0)
Prior vaginal delivery	4/50 (8.0)
Clinical symptoms	
Chronic pelvic pain	47/50 (94.0)
Dysmenorrhea	27/50 (54.0)
Dyspareunia	15/50 (30.0)
Dyschezia	14/50 (28.0)
Abnormal uterine bleeding	10/50 (20.0)
Dysuria	6/50 (12.0)
Obstipation	6/50 (12.0)
Infertility	5/50 (10.0)
Leg pain	5/50 (10.0)
Lower back pain	4/50 (8.0)
Rectal bleeding	3/50 (6.0)
Diarrhea	2/50 (4.0)
Fatigue	2/50 (4.0)
Foot drop	2/50 (4.0)
Leg paresthesia	2/50 (4.0)
Abdominal muscle fasciculations	1/50 (2.0)

### 3.1 MRI image analysis

The percentages of positive #Enzian categorizations assigned in this study among all readers were 24.0% (O), 36.0% (A), 40.0% (B), 33.3% (C), 18.0% (FA), 2.7% (FB), 8.0% (FI), 2.7% (FU) and 6.7% (F(…)).

**Figure 2:**
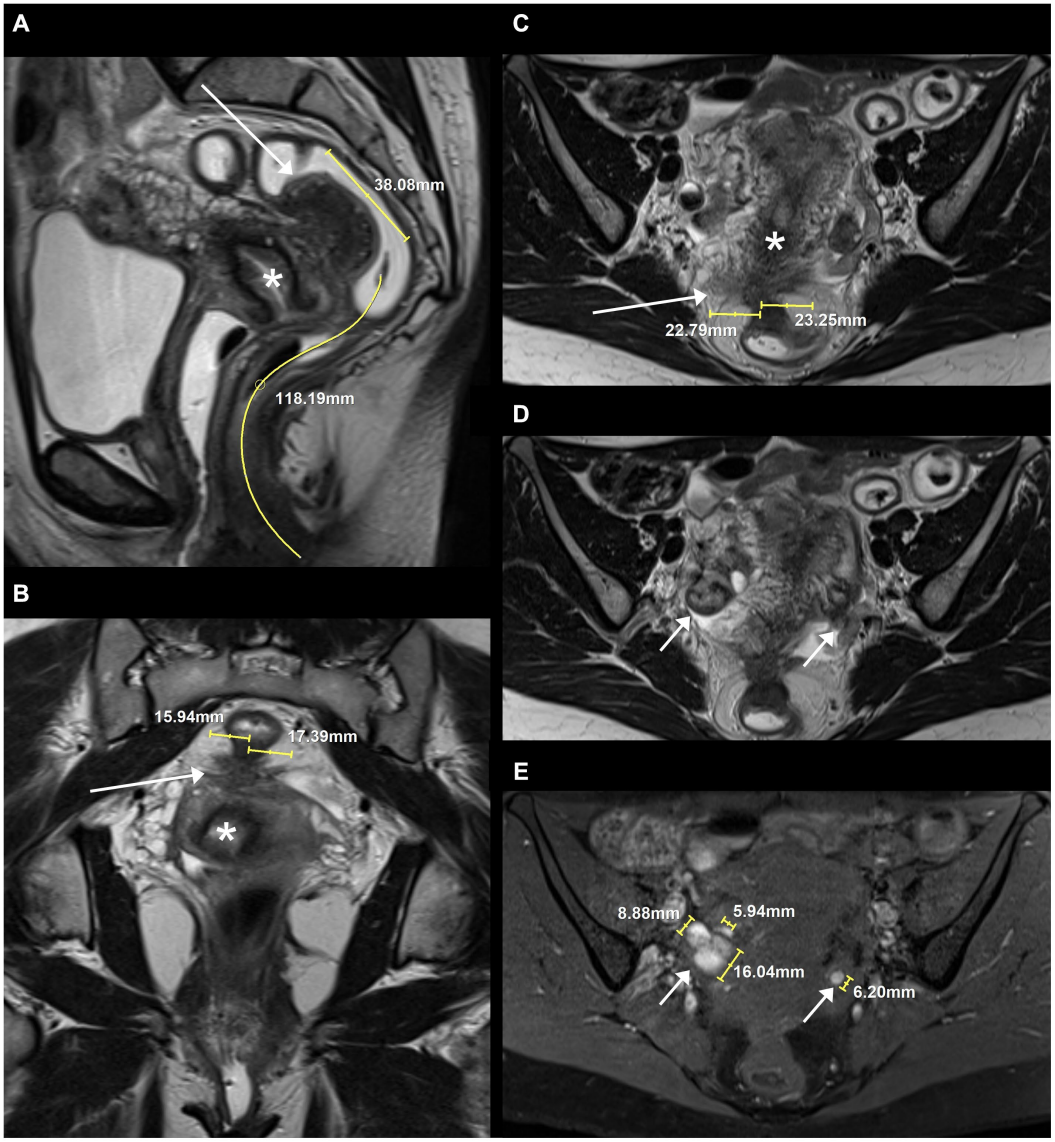
Example of typical deep infiltrating endometriosis (DIE), focal adenomyosis of the outer myometrium and endometriomas on MRI, categorized by two readers as #Enzian(m) O1/2, A2, B2/2, C3, FA and by one reader as #Enzian(m) O1/1, A2, B2/2, C3, FA (due to a borderline summed size of endometriomas on the right side between O1 and O2): **(A)** Sagittal, **(B)** coronal and **(C)** axial T2 FSE (fast spin echo) showing a hypointense mass containing hyperintense foci (long arrows) with extension to rectum, vaginal vault, parametria, and posterior outer myometrium. **(D)** Axial T2 FSE and **(E)** axial fat suppressed T1 FSE demonstrating characteristic bilateral endometriomas with T1w-hyperintensity and T2w-hypointensity (short arrows). Asterisks: cervix uteri.

The agreement between pairs of two readers each are listed in Supplementary Tables S1–S36. An exemplary case of a patient with typical DIE on MRI is shown in Figure 2. Calculations of Cohen’s kappa coefficients (κ) and quadratic weighted kappa coefficients (κ_w_) for pairs of two raters each are presented in Table 2. For the two readers with experience in pelvic MRI (reader 1 and 2), agreement in the assessment of #Enzian categories A, B and C varied from κ = 0.87 (95% CI 0.72–1.00) to κ = 0.96 (95% CI 0.87–1.00) (dichotomized data) and from κ_w_ = 0.84 (95% CI 0.71–0.97) to κ_w_ = 0.89 (95% CI 0.82–0.97) (ordinal data). For the pairings of the readers with experience in pelvic MRI to the reader without specific experience in pelvic MRI (reader 1 and 3, reader 2 and 3), agreement in the assessment of #Enzian categories A, B, and C varied from κ = 0.62 (95% CI 0.39–0.84) to κ = 0.96 (95% CI 0.87–1.00) (dichotomized data) and from κ_w_ = 0.64 (95% CI 0.44–0.85) to κ_w_ = 0.91 (95% CI 0.84–0.98) (ordinal data).

**Table 2:** Agreement of two raters each for the assignment of the #Enzian classification on MRI.

	Reader 1 and 2, 95% CI	Reader 1 and 3, 95% CI	Reader 2 and 3, 95% CI
DIE, all locations^a^	0.92 (0.81–1.00)^*^	0.88 (0.75–1.00)^*^	0.88 (0.75–1.00)^*^
O, dichotomized, both sides^a^	0.95 (0.84–1.00)^*^	0.94 (0.83–1.00)^*^	0.89 (0.74–1.00)^*^
O, dichotomized, left side^a^	1.00 (1.00–1.00)^*^	0.86 (0.68–1.00)^*^	0.86 (0.68–1.00)^*^
O, dichotomized, right side^a^	0.85 (0.65–1.00)^*^	1.00 (1.00–1.00)^*^	0.85 (0.65–1.00)^*^
A, dichotomized^a^	0.96 (0.87–1.00)^*^	0.91 (0.80–1.00)^*^	0.96 (0.87–1.00)^*^
B, dichotomized, both sides^a^	0.92 (0.80–1.00)^*^	0.75 (0.56–0.94)^*^	0.75 (0.56–0.94)^*^
B, dichotomized, left side^a^	0.87 (0.74–1.00)^*^	0.65 (0.44–0.87)^*^	0.62 (0.39–0.84)^*^
B, dichotomized, right side^a^	0.87 (0.72–1.00)^*^	0.69 (0.48–0.90)^*^	0.74 (0.55–0.93)^*^
C, dichotomized^a^	0.87 (0.73–1.00)^*^	0.73 (0.53–0.93)^*^	0.77 (0.58–0.96)^*^
FA^a^	0.61 (0.36.-0.86)^*^	0.39 (0.05.-0.74)^**^	0.37 (0.09.-0.64)^*^
FB^a^	0.66 (0.03.-1.00)^*^	1.00 (1.00–1.00)^*^	0.66 (0.03–1.00)^*^
FU^a^	1.00 (1.00–1.00)^*^	0.00 (0.00–0.00)	0.00 (0.00–0.00)
FI^a^	1.00 (1.00–1.00)^*^	0.55 (0.10–0.99)^*^	0.55 (0.10–0.99)^*^
F(…)^a^	0.88 (0.64–1.00)^*^	0.31 (−0.16–0.78)^**^	0.38 (−0.15–0.91)^*^
O, 0–3, left side^b^	1.00 (1.00–1.00)^*^	0.91 (0.82–0.99)^*^	0.91 (0.82–0.99)^*^
O, 0–3, right side^b^	0.96 (0.89–1.00)^*^	0.96 (0.91–1.00)^*^	0.92 (0.84–1.00)^*^
A, 0–3^b^	0.84 (0.71–0.97)^*^	0.89 (0.80–0.97)^*^	0.91 (0.84–0.98)^*^
B, 0–3, left side^b^	0.88 (0.79–0.97)^*^	0.66 (0.47–0.84)^*^	0.64 (0.44–0.85)^*^
B, 0–3, right side^b^	0.89 (0.80–0.98)^*^	0.67 (0.49–0.85)^*^	0.70 (0.53–0.87)^*^
C, 0–3^b^	0.89 (0.82–0.97)^*^	0.83 (0.70–0.97)^*^	0.89 (0.78–1.00)^*^

DIE, deep infiltrating endometriosis. ^a^Cohen’s kappa. ^b^Weighted kappa, quadratic weights. ^*^p < 0.001. ^**^p = 0.002.

Readers 1 and 2 had 5–7 years’ experience in endometriosis MRI, reader 3 had no experience and received a 1 h training.

Calculations of Fleiss’ kappa and Kendall’s W for the ratings of all three readers are shown in Table 3. For #Enzian categories A, B, and C, Fleiss’ kappa varied from 0.72 (95% CI 0.56–0.88) to 0.94 (95% CI 0.78–1.00) (dichotomized data) and Kendall’s W from 0.84 to 0.96 (ordinal data).

**Table 3:** Agreement of three raters for the assignment of the #Enzian classification on MRI.

	Reader 1, 2 and 3, 95% CI
DIE, all locations^a^	0.89 (0.73–1.00)^*^
O, dichotomized, both sides^a^	0.93 (0.77–1.00)^*^
O, dichotomized, left side^a^	0.91 (0.75–1.00)^*^
O, dichotomized, right side^a^	0.90 (0.74–1.00)^*^
A, dichotomized^a^	0.94 (0.78–1.00)^*^
B, dichotomized, both sides^a^	0.81 (0.65–0.97)^*^
B, dichotomized, left side^a^	0.72 (0.56–0.88)^*^
B, dichotomized, right side^a^	0.77 (0.61–0.93)^*^
C, dichotomized^a^	0.79 (0.63–0.95)^*^
FA^a^	0.46 (0.30–0.62)^*^
FB^a^	0.74 (0.58–0.90)^*^
FU^a^	0.49 (0.33–0.65)^*^
FI^a^	0.73 (0.57–0.89)^*^
F(…)^a^	0.57 (0.41–0.73)^*^
O, 0–3, left side^b^	0.95^*^
O, 0–3, right side^b^	0.95^*^
A, 0–3^b^	0.96^*^
B, 0–3, left side^b^	0.84^*^
B, 0–3, right side^b^	0.86^*^
C, 0–3^b^	0.90^*^

DIE, deep infiltrating endometriosis. ^a^Fleiss’ kappa. ^b^Kendall W. ^*^p < 0.001.

Findings for category F(…) were concordantly noted by three readers in one case, where DIE was located in the anterior abdominal wall (intramuscular). Two of three readers reported DIE in single cases in the sciatic nerve, inguinal canal, and sacral plexus, respectively. In one case, only one of the three readers diagnosed DIE affecting the anterior abdominal wall (subcutaneous).

Calculations of Cohen’s kappa coefficients (κ) and quadratic weighted kappa coefficients (κ_w_) for the two assessments of reader 1 are presented in Table 4.

**Table 4:** Intraobserver agreement for the assignment of the #Enzian classification on MRI.

	Reader 1, 95% CI
DIE, all locations^a^	0.96 (0.88–1.00)^*^
O, dichotomized, both sides^a^	1.00 (1.00–1.00)^*^
O, dichotomized, left side^a^	1.00 (1.00–1.00)^*^
O, dichotomized, right side^a^	0.92 (0.77–1.00)^*^
A, dichotomized^a^	0.86 (0.72–1.00)^*^
B, dichotomized, both sides^a^	0.96 (0.88–1.00)^*^
B, dichotomized, left side^a^	0.87 (0.73–1.00)^*^
B, dichotomized, right side^a^	0.91 (0.79–1.00)^*^
C, dichotomized^a^	0.96 (0.87–1.00)^*^
FA^a^	0.82 (0.63–1.00)^*^
FB^a^	1.00 (1.00.-1.00)^*^
FU^a^	1.00 (1.00–1.00)^*^
FI^a^	0.88 (0.64–1.00)^*^
F(…)^a^	0.88 (0.64–1.00)^*^
O, 0–3, left side^b^	1.00 (1.00–1.00)^*^
O, 0–3, right side^b^	0.95 (0.90–1.00)^*^
A, 0–3^b^	0.89 (0.80–0.97)^*^
B, 0–3, left side^b^	0.85 (0.73–0.96)^*^
B, 0–3, right side^b^	0.90 (0.84–0.97)^*^
C, 0–3^b^	0.95 (0.89–1.00)^*^

DIE, deep infiltrating endometriosis. ^a^Cohen’s kappa. ^b^Weighted kappa, quadratic weights. ^*^p < 0.001.

## 4 Discussion

In our study, we prospectively evaluated inter- and intraobserver agreement of the MRI-applicable categories of the 2021 #Enzian-classification for endometriosis through a total of 50 MRI cases assessed by three readers from two different institutions. Our study demonstrated overall excellent interobserver agreement of the assessments of three independent readers for the diagnosis of deep infiltrating endometriosis on MRI with a Fleiss’ kappa of 0.89 (95% CI 0.73–1.00), and for the diagnosis of endometriomas on MRI with a Fleiss’ kappa of 0.93 (95% CI 0.77–1.00). Only moderate interobserver agreement was found in the evaluation of uterine adenomyosis, with a Fleiss’ kappa of 0.46 (95% CI 0.30–0.62). Intraobserver agreement was excellent for all evaluated categories of the #Enzian classification. Our study indicated that radiologists without specific experience in pelvic MRI can achieve substantial to excellent agreement with experienced radiologists in the application of the #Enzian classification on MRI after only a short training and with guidance from explanatory illustrations.

To our knowledge, this is the first study to prospectively evaluate interobserver agreement of the MRI-based application of the 2021 #Enzian classification, in which endometriomas (O0-3, separately for the left and right body side) and separate category B values (B0-3) for the left and right body side were included. In addition, and in contrast to previous studies, we performed analyses of the non-dichotomized, ordinal scaled data as specified in the classification. The only other study to date on interobserver variability of the updated #Enzian classification is the 2021 study by Manganaro et al. In their retrospective analysis of 60 cases, excellent interobserver agreement was stated for the diagnosis of endometriomas (κ: 0.8153) and good agreement for the assessments of compartments/categories A (κ: 0.7645), B (κ: 0.74023), C (κ: 0.7932) and F (extragenital deep infiltrating endometriosis, κ: 0.6349) (12). However, results of a separate evaluation of endometriomas and compartment B by body side and individual results for categories FA, FB, FI, FU and F(…) were not reported. In addition, no weighted kappa values were reported for the ordinally scaled data in categories A, B, C, and O. However, this detailed information is of importance because a difference of one grade (e.g., B3 versus B2) is less significant in practice than a difference of several grades (e.g., B3 versus B0). This is taken into account in our study with the analysis of quadratically weighted kappa values. Finally, as mentioned above, it is also important in practice whether the intended separate description of category B and O findings by body side can be correctly performed on MRI images using the #Enzian classification. Our results suggest that the side-separated description of findings is useful and feasible, but also confirm the observation of other authors that assessment in category B can be challenging on MRI (10). However, it is inherent in the design of the classification that it is not a matter of an exact size measurement, but rather of a category assignment (1: < 1 cm, 2: 1–3 cm, 3: > 3 cm; see exemplary Figures 2B, C).

Several studies retrospectively evaluated interobserver agreement of the 2011 Enzian classification for the MRI-based diagnosis of DIE, obtaining varying results. Thomassin-Naggara et al. reported excellent agreement for category C (κ 0.88, 95% CI 0.82–0.94), good agreement for category A (κ 0.79, 95% CI 0.67–0.9) and poor agreement for category B (κ 0.41, 95% CI 0.26–0.56) (*n* = 150) (21). Thus, greater difficulties were noted by the authors in the assessment of Enzian category B on MRI compared to categories A and C. In contrast to Thomassin-Naggara et al. we found excellent interobserver variability for the experienced readers as well as excellent intraobserver variability for category B.

In a previous study (3), we also found excellent agreement for category C (κ_w_ 0.89, 95% CI: 0.75–1.00), but moderate agreement for category A (κ_w_ 0.57, 95% CI: 0.13–1.00) and category B (κ_w_ 0.44, 95% CI: 0.11–0.76) (*n* = 20), although the smaller number of cases and also the adjustment effect in the application of the classification by two of the readers from the previous to the present study must be taken into account. No more than fair agreement of three radiologists was found by Burla et al. in their 2021 study (κ 0.255 for category A, 0.146 for category B, −0.263 for category C) (*n* = 23) (22). Previous studies also concluded that agreement in the detection of DIE at the uterosacral ligaments between different readers is not optimal (23, 24), an observation that we cannot currently confirm when considering the agreement of the two experienced readers.

**Figure 3:**
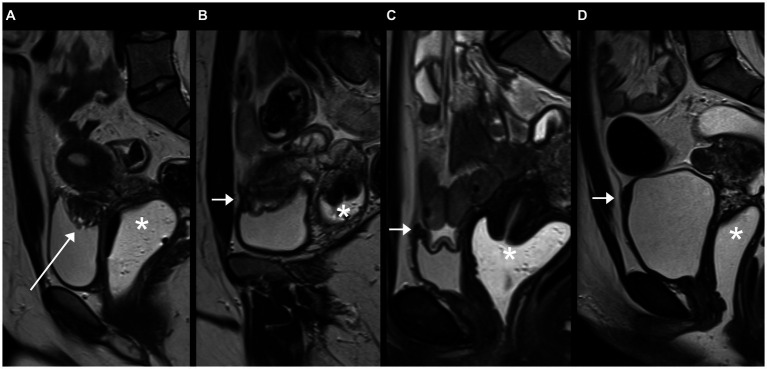
Sagittal T2 FSE (fast spin echo) slices demonstrating bladder findings of different patients on MRI: **(A)** Typical deep infiltrating endometriosis (DIE) of the bladder dome (hypointense mass with hyperintense foci, long arrow), rated #Enzian FB by all three readers, **(B)** focal thickening of the anterior bladder wall (short arrow), scored #Enzian FB by one of three readers due to off-midline location and central T2w-hyperintensity (no endometriosis on laparoscopy), **(C)** and **(D)** focal thickening of the anterior bladder wall (short arrows), interpreted as prominent urachal remnant by all three readers due to midline location on the serosal surface and the presence of a thin band, extending from the bladder dome toward the umbilicus. Asterisks: vaginal vault.

Various groups have provided definitions of the appearance of endometriosis on MRI (25–27) and recently, a structured report template based on the #Enzian classification has been provided by Maciel et al. (10). Figure 3 demonstrates on the example of the urinary bladder how certain discrepancies in the agreement of several readers can occur on the verge of normal and pathological (28). Similar diagnostic challenges have led to the only moderate agreement in the diagnosis of adenomyosis (#Enzian FA) in our study, which can be mimicked by uterine contractions and for which diagnostic criteria on MRI are not without controversy (17, 29).

Further efforts to educate radiologists in endometriosis diagnostics are desirable to improve reliability of readings, as several studies underscore: Saba et al. found that the accuracy of MRI diagnosis of endometriosis increased with radiologist experience when the same cases were reanalyzed after 12 and 24 months by the same reader (13). Jaramillo-Cardoso stated in their 2019 study that a structured expert-read outperformed routine reads and structured reported reads of pelvic MRIs for endometriosis, considering sensitivity and specificity and using surgical staging as reference (30). A 1 h training session and the provision of explanatory illustrations enabled a previously inexperienced radiologist to achieve remarkable agreement to experienced radiologists in our study.

Despite the explained strengths of this study, the conduction in a single tertiary care center might be a limitation of our study, whereby radiologists from two different institutions performed the analysis. When viewed in conjunction with our previous and other studies, the study population is typical of an endometriosis center, with relatively high rates of patients who had prior surgeries and patients presenting with infertility. Further studies on the reliability and validity of the #Enzian classification are desirable. The comparison of MRI assessments using the #Enzian classification with results of surgical procedures was not the subject of this study but should also be prospectively investigated in further studies to expand on the literature in this regard (31), considering a separate analysis by body side.

In conclusion, the #Enzian classification enables the achievement of excellent inter- and intraobserver agreement in MRI-based diagnosis of deep infiltrating endometriosis and endometriomas with sufficient reader experience. The #Enzian classification could be recommended for routine use by radiologists in daily pelvic MRI scans for endometriosis.

## Data availability statement

The raw data supporting the conclusions of this article will be made available by the authors, without undue reservation.

## Ethics statement

The studies involving humans were approved by Institutional Review Board of the Faculty of Medicine, Justus-Liebig-University Giessen. The studies were conducted in accordance with the local legislation and institutional requirements. The participants provided their written informed consent to participate in this study.

## Author contributions

SH: Conceptualization, Data curation, Formal analysis, Investigation, Methodology, Project administration, Visualization, Writing – original draft. HK: Data curation, Investigation, Writing – review & editing. FZ: Conceptualization, Methodology, Resources, Writing – review & editing. IM-H: Conceptualization, Methodology, Resources, Writing – review & editing. JK: Conceptualization, Supervision, Writing – review & editing. SY: Data curation, Methodology, Writing – review & editing. EN: Data curation, Methodology, Writing – review & editing. GK: Conceptualization, Methodology, Project administration, Supervision, Writing – review & editing. FR: Conceptualization, Investigation, Methodology, Project administration, Supervision, Validation, Writing – review & editing.
