# Supplementary material for: Application of the #Enzian classification for endometriosis on MRI: prospective evaluation of inter- and intraobserver agreement
Source: Front Med (Lausanne). 2023 Nov 17;10:1303593. doi: 10.3389/fmed.2023.1303593 (PMC10690940; doi:10.3389/fmed.2023.1303593)
Supplement: Supplementary file 1 [file Table_1.DOCX]

Supplementary Material

# Supplementary Tables

Supplementary Table S1 Pairwise comparison of DIE-diagnosis, all locations, reader 1 and 2

Supplementary Table S2 Pairwise comparison of DIE-diagnosis, all locations, reader 1 and 3

Supplementary Table S3 Pairwise comparison of DIE-diagnosis, all locations, reader 2 and 3

Supplementary Table S4 Pairwise comparison of assignments for #Enzian category O left side, reader 1 and 2

Supplementary Table S5 Pairwise comparison of assignments for #Enzian category O left side, reader 1 and 3

Supplementary Table S6 Pairwise comparison of assignments for #Enzian category O left side, reader 2 and 3

Supplementary Table S7 Pairwise comparison of assignments for #Enzian category O right side, reader 1 and 2

Supplementary Table S8 Pairwise comparison of assignments for #Enzian category O right side, reader 1 and 3

Supplementary Table S9 Pairwise comparison of assignments for #Enzian category O right side, reader 2 and 3

Supplementary Table S10 Pairwise comparison of assignments for #Enzian category A, reader 1 and 2

Supplementary Table S11 Pairwise comparison of assignments for #Enzian category A, reader 1 and 3

Supplementary Table S12 Pairwise comparison of assignments for #Enzian category A, reader 2 and 3

Supplementary Table S13 Pairwise comparison of assignments for #Enzian category B left side, reader 1 and 2

Supplementary Table S14 Pairwise comparison of assignments for #Enzian category B left side, reader 1 and 3

Supplementary Table S15 Pairwise comparison of assignments for #Enzian category B left side, reader 2 and 3

Supplementary Table S16 Pairwise comparison of assignments for #Enzian category B right side, reader 1 and 2

Supplementary Table S17 Pairwise comparison of assignments for #Enzian category B right side, reader 1 and 3

Supplementary Table S18 Pairwise comparison of assignments for #Enzian category B right side, reader 2 and 3

Supplementary Table S19 Pairwise comparison of assignments for #Enzian category C, reader 1 and 2

Supplementary Table S20 Pairwise comparison of assignments for #Enzian category C, reader 1 and 3

Supplementary Table S21 Pairwise comparison of assignments for #Enzian category C, reader 2 and 3

Supplementary Table S22 Pairwise comparison of assignments for #Enzian category FA, reader 1 and 2

Supplementary Table S23 Pairwise comparison of assignments for #Enzian category FA, reader 1 and 3

Supplementary Table S24 Pairwise comparison of assignments for #Enzian category FA, reader 2 and 3

Supplementary Table S25 Pairwise comparison of assignments for #Enzian category FB, reader 1 and 2

Supplementary Table S26 Pairwise comparison of assignments for #Enzian category FB, reader 1 and 3

Supplementary Table S27 Pairwise comparison of assignments for #Enzian category FB, reader 2 and 3

Supplementary Table S28 Pairwise comparison of assignments for #Enzian category FU, reader 1 and 2

Supplementary Table S29 Pairwise comparison of assignments for #Enzian category FU, reader 1 and 3

Supplementary Table S30 Pairwise comparison of assignments for #Enzian category FU, reader 2 and 3

Supplementary Table S31 Pairwise comparison of assignments for #Enzian category FI, reader 1 and 2

Supplementary Table S32 Pairwise comparison of assignments for #Enzian category FI, reader 1 and 3

Supplementary Table S33 Pairwise comparison of assignments for #Enzian category FI, reader 2 and 3

Supplementary Table S34 Pairwise comparison of assignments for #Enzian category F(…), reader 1 and 2

Supplementary Table S35 Pairwise comparison of assignments for #Enzian category F(…), reader 1 and 3

Supplementary Table S36 Pairwise comparison of assignments for #Enzian category F(…), reader 2 and 3

**Supplementary Table S1. Pairwise comparison of DIE-diagnosis, all locations, reader 1 and 2**

|  | Reader 2 | |
| --- | --- | --- |
| Reader 1 | DIE- | DIE+ |
| DIE- | 26 | 0 |
| DIE+ | 2 | 22 |

**Supplementary Table S2. Pairwise comparison of DIE-diagnosis, all locations, reader 1 and 3**

|  | Reader 3 | |
| --- | --- | --- |
| Reader 1 | DIE- | DIE+ |
| DIE- | 24 | 2 |
| DIE+ | 1 | 23 |

**Supplementary Table S3. Pairwise comparison of DIE-diagnosis, all locations, reader 2 and 3**

|  | Reader 3 | |
| --- | --- | --- |
| Reader 2 | DIE- | DIE+ |
| DIE- | 25 | 3 |
| DIE+ | 0 | 22 |

**Supplementary Table S4. Pairwise comparison of assignments for #Enzian category O left side, reader 1 and 2**

|  | Reader 2 | |  |  |
| --- | --- | --- | --- | --- |
| Reader 1 | O0, left side | O1, left side | O2, left side | O3, left side |
| O0, left side | 41 | 0 | 0 | 0 |
| O1, left side | 0 | 5 | 0 | 0 |
| O2, left side | 0 | 0 | 3 | 0 |
| O3, left side | 0 | 0 | 0 | 1 |

**Supplementary Table S5. Pairwise comparison of assignments for #Enzian category O left side, reader 1 and 3**

|  | Reader 3 | |  |  |
| --- | --- | --- | --- | --- |
| Reader 1 | O0, left side | O1, left side | O2, left side | O3, left side |
| O0, left side | 40 | 1 | 0 | 0 |
| O1, left side | 1 | 3 | 1 | 0 |
| O2, left side | 0 | 0 | 3 | 0 |
| O3, left side | 0 | 0 | 1 | 0 |

**Supplementary Table S6. Pairwise comparison of assignments for #Enzian category O left side, reader 2 and 3**

|  | Reader 3 | |  |  |
| --- | --- | --- | --- | --- |
| Reader 2 | O0, left side | O1, left side | O2, left side | O3, left side |
| O0, left side | 40 | 1 | 0 | 0 |
| O1, left side | 1 | 3 | 1 | 0 |
| O2, left side | 0 | 0 | 3 | 0 |
| O3, left side | 0 | 0 | 1 | 0 |

**Supplementary Table S7. Pairwise comparison of assignments for #Enzian category O right side, reader 1 and 2**

|  | Reader 2 | |  |  |
| --- | --- | --- | --- | --- |
| Reader 1 | O0, right side | O1, right side | O2, right side | O3, right side |
| O0, right side | 41 | 2 | 0 | 0 |
| O1, right side | 0 | 2 | 0 | 0 |
| O2, right side | 0 | 0 | 4 | 0 |
| O3, right side | 0 | 0 | 0 | 1 |

**Supplementary Table S8. Pairwise comparison of assignments for #Enzian category O right side, reader 1 and 3**

|  | Reader 3 | |  |  |
| --- | --- | --- | --- | --- |
| Reader 1 | O0, right side | O1, right side | O2, right side | O3, right side |
| O0, right side | 43 | 0 | 0 | 0 |
| O1, right side | 0 | 2 | 0 | 0 |
| O2, right side | 0 | 1 | 2 | 1 |
| O3, right side | 0 | 0 | 0 | 1 |

**Supplementary Table S9. Pairwise comparison of assignments for #Enzian category O right side, reader 2 and 3**

|  | Reader 3 | |  |  |
| --- | --- | --- | --- | --- |
| Reader 2 | O0, right side | O1, right side | O2, right side | O3, right side |
| O0, right side | 41 | 0 | 0 | 0 |
| O1, right side | 2 | 2 | 0 | 0 |
| O2, right side | 0 | 1 | 2 | 1 |
| O3, right side | 0 | 0 | 0 | 1 |

**Supplementary Table S10. Pairwise comparison of assignments for #Enzian category A, reader 1 and 2**

|  | Reader 2 | |  |  |
| --- | --- | --- | --- | --- |
| Reader 1 | A0 | A1 | A2 | A3 |
| A0 | 32 | 0 | 1 | 0 |
| A1 | 0 | 5 | 6 | 0 |
| A2 | 0 | 0 | 6 | 0 |
| A3 | 0 | 0 | 0 | 0 |

**Supplementary Table S11. Pairwise comparison of assignments for #Enzian category A, reader 1 and 3**

|  | Reader 3 | |  |  |
| --- | --- | --- | --- | --- |
| Reader 1 | A0 | A1 | A2 | A3 |
| A0 | 31 | 2 | 0 | 0 |
| A1 | 0 | 8 | 3 | 0 |
| A2 | 0 | 1 | 5 | 0 |
| A3 | 0 | 0 | 0 | 0 |

**Supplementary Table S12. Pairwise comparison of assignments for #Enzian category A, reader 2 and 3**

|  | Reader 3 | |  |  |
| --- | --- | --- | --- | --- |
| Reader 2 | A0 | A1 | A2 | A3 |
| A0 | 31 | 1 | 0 | 0 |
| A1 | 0 | 5 | 0 | 0 |
| A2 | 0 | 5 | 8 | 0 |
| A3 | 0 | 0 | 0 | 0 |

**Supplementary Table S13. Pairwise comparison of assignments for #Enzian category B left side, reader 1 and 2**

|  | Reader 2 | |  |  |
| --- | --- | --- | --- | --- |
| Reader 1 | B0, left side | B1, left side | B2, left side | B3, left side |
| B0, left side | 29 | 2 | 0 | 0 |
| B1, left side | 0 | 0 | 0 | 0 |
| B2, left side | 1 | 3 | 12 | 0 |
| B3, left side | 0 | 0 | 3 | 0 |

**Supplementary Table S14. Pairwise comparison of assignments for #Enzian category B left side, reader 1 and 3**

|  | Reader 3 | |  |  |
| --- | --- | --- | --- | --- |
| Reader 1 | B0, left side | B1, left side | B2, left side | B3, left side |
| B0, left side | 28 | 3 | 0 | 0 |
| B1, left side | 0 | 0 | 0 | 0 |
| B2, left side | 5 | 7 | 4 | 0 |
| B3, left side | 0 | 0 | 2 | 1 |

**Supplementary Table S15. Pairwise comparison of assignments for #Enzian category B left side, reader 2 and 3**

|  | Reader 3 | |  |  |
| --- | --- | --- | --- | --- |
| Reader 2 | B0, left side | B1, left side | B2, left side | B3, left side |
| B0, left side | 27 | 3 | 0 | 0 |
| B1, left side | 2 | 3 | 0 | 0 |
| B2, left side | 4 | 4 | 6 | 1 |
| B3, left side | 0 | 0 | 0 | 0 |

**Supplementary Table S16. Pairwise comparison of assignments for #Enzian category B right side, reader 1 and 2**

|  | Reader 2 | |  |  |
| --- | --- | --- | --- | --- |
| Reader 1 | B0, right side | B1, right side | B2, right side | B3, right side |
| B0, right side | 31 | 2 | 0 | 0 |
| B1, right side | 0 | 1 | 0 | 0 |
| B2, right side | 1 | 3 | 8 | 1 |
| B3, right side | 0 | 0 | 1 | 2 |

**Supplementary Table S17. Pairwise comparison of assignments for #Enzian category B right side, reader 1 and 3**

|  | Reader 3 | |  |  |
| --- | --- | --- | --- | --- |
| Reader 1 | B0, right side | B1, right side | B2, right side | B3, right side |
| B0, right side | 29 | 3 | 1 | 0 |
| B1, right side | 0 | 1 | 0 | 0 |
| B2, right side | 3 | 6 | 4 | 0 |
| B3, right side | 0 | 0 | 3 | 0 |

**Supplementary Table S18. Pairwise comparison of assignments for #Enzian category B right side, reader 2 and 3**

|  | Reader 3 | |  |  |
| --- | --- | --- | --- | --- |
| Reader 2 | B0, right side | B1, right side | B2, right side | B3, right side |
| B0, right side | 29 | 2 | 1 | 0 |
| B1, right side | 1 | 4 | 1 | 0 |
| B2, right side | 2 | 4 | 3 | 0 |
| B3, right side | 0 | 0 | 3 | 0 |

**Supplementary Table S19. Pairwise comparison of assignments for #Enzian category C, reader 1 and 2**

|  | Reader 2 | |  |  |
| --- | --- | --- | --- | --- |
| Reader 1 | C0 | C1 | C2 | C3 |
| C0 | 30 | 1 | 0 | 0 |
| C1 | 2 | 5 | 5 | 0 |
| C2 | 0 | 0 | 4 | 1 |
| C3 | 0 | 0 | 0 | 2 |

**Supplementary Table S20. Pairwise comparison of assignments for #Enzian category C, reader 1 and 3**

|  | Reader 3 | |  |  |
| --- | --- | --- | --- | --- |
| Reader 1 | C0 | C1 | C2 | C3 |
| C0 | 31 | 0 | 0 | 0 |
| C1 | 5 | 4 | 3 | 0 |
| C2 | 1 | 0 | 3 | 1 |
| C3 | 0 | 0 | 0 | 2 |

**Supplementary Table S21. Pairwise comparison of assignments for #Enzian category C, reader 2 and 3**

|  | Reader 3 | |  |  |
| --- | --- | --- | --- | --- |
| Reader 2 | C0 | C1 | C2 | C3 |
| C0 | 32 | 0 | 0 | 0 |
| C1 | 4 | 2 | 0 | 0 |
| C2 | 1 | 2 | 6 | 0 |
| C3 | 0 | 0 | 0 | 3 |

**Supplementary Table S22. Pairwise comparison of assignments for #Enzian category FA, reader 1 and 2**

|  | Reader 2 | |
| --- | --- | --- |
| Reader 1 | FA- | FA+ |
| FA- | 35 | 6 |
| FA+ | 1 | 8 |

**Supplementary Table S23. Pairwise comparison of assignments for #Enzian category FA, reader 1 and 3**

|  | Reader 3 | |
| --- | --- | --- |
| Reader 1 | FA- | FA+ |
| FA- | 40 | 1 |
| FA+ | 6 | 3 |

**Supplementary Table S24. Pairwise comparison of assignments for #Enzian category FA, reader 2 and 3**

|  | Reader 3 | |
| --- | --- | --- |
| Reader 2 | FA- | FA+ |
| FA- | 36 | 0 |
| FA+ | 10 | 4 |

**Supplementary Table S25. Pairwise comparison of assignments for #Enzian category FB, reader 1 and 2**

|  | Reader 2 | |
| --- | --- | --- |
| Reader 1 | FB- | FB+ |
| FB- | 48 | 1 |
| FB+ | 0 | 1 |

**Supplementary Table S26. Pairwise comparison of assignments for #Enzian category FB, reader 1 and 3**

|  | Reader 3 | |
| --- | --- | --- |
| Reader 1 | FB- | FB+ |
| FB- | 49 | 0 |
| FB+ | 0 | 1 |

**Supplementary Table S27. Pairwise comparison of assignments for #Enzian category FB, reader 2 and 3**

|  | Reader 3 | |
| --- | --- | --- |
| Reader 2 | FB- | FB+ |
| FB- | 48 | 0 |
| FB+ | 1 | 1 |

**Supplementary Table S28. Pairwise comparison of assignments for #Enzian category FU, reader 1 and 2**

|  | Reader 2 | |
| --- | --- | --- |
| Reader 1 | FU- | FU+ |
| FU- | 48 | 0 |
| FU+ | 0 | 2 |

**Supplementary Table S29. Pairwise comparison of assignments for #Enzian category FU, reader 1 and 3**

|  | Reader 3 | |
| --- | --- | --- |
| Reader 1 | FU- | FU+ |
| FU- | 48 | 0 |
| FU+ | 2 | 0 |

**Supplementary Table S30. Pairwise comparison of assignments for #Enzian category FU, reader 2 and 3**

|  | Reader 3 | |
| --- | --- | --- |
| Reader 2 | FU- | FU+ |
| FU- | 48 | 0 |
| FU+ | 2 | 0 |

**Supplementary Table S31. Pairwise comparison of assignments for #Enzian category FI, reader 1 and 2**

|  | Reader 2 | |
| --- | --- | --- |
| Reader 1 | FI- | FI+ |
| FI- | 45 | 0 |
| FI+ | 0 | 5 |

**Supplementary Table S32. Pairwise comparison of assignments for #Enzian category FI, reader 1 and 3**

|  | Reader 3 | |
| --- | --- | --- |
| Reader 1 | FI- | FI+ |
| FI- | 45 | 0 |
| FI+ | 3 | 2 |

**Supplementary Table S33. Pairwise comparison of assignments for #Enzian category FI, reader 2 and 3**

|  | Reader 3 | |
| --- | --- | --- |
| Reader 2 | FI- | FI+ |
| FI- | 45 | 0 |
| FI+ | 3 | 2 |

**Supplementary Table S34. Pairwise comparison of assignments for #Enzian category F(…), reader 1 and 2**

|  | Reader 2 | |
| --- | --- | --- |
| Reader 1 | F(…)- | F(…)+ |
| F(…)- | 45 | 0 |
| F(…)+ | 1 | 4 |

**Supplementary Table S35. Pairwise comparison of assignments for #Enzian category F(…), reader 1 and 3**

|  | Reader 3 | |
| --- | --- | --- |
| Reader 1 | F(…)- | F(…)+ |
| F(…)- | 45 | 0 |
| F(…)+ | 4 | 1 |

**Supplementary Table S36. Pairwise comparison of assignments for #Enzian category F(…), reader 2 and 3**

|  | Reader 3 | |
| --- | --- | --- |
| Reader 2 | F(…)- | F(…)+ |
| F(…)- | 46 | 0 |
| F(…)+ | 3 | 1 |
